# The Effects of C75, an Inhibitor of Fatty Acid Synthase, on Sleep and Metabolism in Mice

**DOI:** 10.1371/journal.pone.0030651

**Published:** 2012-02-13

**Authors:** Jacob Pellinen, Éva Szentirmai

**Affiliations:** 1 Washington, Wyoming, Alaska, Montana and Idaho (WWAMI) Medical Education Program, Washington State University, Spokane, Washington, United States of America; 2 Department of Veterinary and Comparative Anatomy, Pharmacology and Physiology, Washington State University, Spokane, Washington, United States of America; 3 Sleep and Performance Research Center, Washington State University, Spokane, Washington, United States of America; Institut Pluridisciplinaire Hubert Curien, France

## Abstract

Sleep is greatly affected by changes in metabolic state. A possible mechanism where energy-sensing and sleep-regulatory functions overlap is related to lipid metabolism. Fatty acid synthase (FAS) plays a central role in lipid metabolism as a key enzyme in the formation of long-chain fatty acids. We studied the effects of systemic administration of C75, an inhibitor of FAS, on sleep, behavioral activity and metabolic parameters in mice. Since the effects of C75 on feeding and metabolism are the opposite of ghrelin's and C75 suppresses ghrelin production, we also tested the role of ghrelin signaling in the actions of C75 by using ghrelin receptor knockout (KO) mice. After a transient increase in wakefulness, C75 elicited dose-dependent and long lasting inhibition of REMS, motor activity and feeding. Simultaneously, C75 significantly attenuated slow-wave activity of the electroencephalogram. Energy expenditure, body temperature and respiratory exchange ratio were suppressed. The diurnal rhythm of feeding was completely abolished by C75. There was significant correlation between the anorectic effects, the decrease in motor activity and the diminished energy expenditure after C75 injection. We found no significant difference between wild-type and ghrelin receptor KO mice in their sleep and metabolic responses to C75. The effects of C75 resemble to what was previously reported in association with visceral illness. Our findings suggest that sleep and metabolic effects of C75 in mice are independent of the ghrelin system and may be due to its aversive actions in mice.

## Introduction

There is a known relationship among feeding, metabolism and sleep. In mice and rats, metabolic states characterized by positive energy balance are typically associated with increased sleep while food deprivation stimulates wakefulness and motor activity. There is a positive correlation between meal size and the length of the subsequent sleep period in rats [1]. Increased feeding induced by prior food deprivation elicits postprandial sleep [2]–[4]. Ventromedial hypothalamic (VMH) lesion [5] is associated with increased sleep time in rats. Increased adiposity induced by cafeteria diet or [6] high-fat diet [7] and obesity due to leptin [8] or leptin receptor [9] deficiency also leads to increases in sleep. Satiety and adiposity signals are likely to mediate the effects of positive energy states on sleep. Satiety hormones, such as cholecystokinin (CCK) [10]–[12] and insulin [13], [14] are released postprandially and stimulate sleep. Hormones of the adipose tissue, such as leptin [15] and tumor necrosis factor (TNF) [16], [17], also enhance sleep and suppress feeding. Postprandial sleep is prevented by CCK1 receptor antagonists [3].

A possible focal point where the regulation of feeding and sleep may converge is lipid metabolism. Fatty acid synthase (FAS) plays a central role in lipid metabolism as a key enzyme in the formation of long-chain fatty acids (reviewed in [18]). The role of FAS in sleep regulation has not been studied. Since the activity of the enzyme is enhanced in anabolic states, it is possible that postprandial and obesity-associated enhanced sleep is related to increased FAS activity. To gain insight into the potential role of FAS in sleep regulation, we tested the effects of C75, an irreversible FAS inhibitor [19]–[21], on sleep in mice.

Ghrelin has been shown to play a role in arousal responses to fasting [22]. Ghrelin is a 28-amino acid peptide, produced by the stomach and hypothalamic neurons (reviewed in [23]). It is the endogenous ligand of the growth hormone secretagogue receptor 1a (GHSR 1a, ghrelin receptor; [24]). Ghrelin receptors are expressed by various brain regions, such as the arcuate nucleus (ARC), lateral hypothalamus (LH), VMH and suprachiasmatic nucleus (SCN), structures known to be involved in feeding and sleep regulation [25]–[27]. Ghrelin secretion is stimulated by fasting and ghrelin enhances feeding and increases adiposity in rats [28], [29]. Growing body of evidence indicates that ghrelin signaling plays a role in the function of arousal mechanisms. Systemic, intracerebroventricular (icv) or intrahypothalamic administration of ghrelin suppresses sleep [30]–[32] in rats. Ghrelin receptor KO mice show attenuated arousal responses to food deprivation and to the exposure of novel environment [22]. Ghrelin is also implicated in the function of thermoregulatory mechanisms and in the integration of sleep and thermoregulatory responses. Central administration of ghrelin diminishes the activity of brown adipose tissue, a key effector organ in non-shivering thermogenesis, by suppressing the activity of its sympathetic innervation [33], [34]. The product(s) of the preproghrelin gene play a role in coordinating thermoregulatory/metabolic and sleep responses to metabolic challenges. When fasted in the cold, normal mice develop hypothermic (torpor) bouts and increased sleep during these hypothermic periods. Ghrelin deficient preproghrelin knockout (KO) mice are incapable of mounting sleep responses under these conditions and enter precipitous, lethal, hypothermia [35].

FAS inhibitors, such as C75 greatly suppress ghrelin production by the stomach and the hypothalamus [36]. C75 potently suppresses eating [37] and energy expenditure [38], [39]. Since ghrelin stimulates feeding and transgenic mice with elevated circulating ghrelin levels have increased energy expenditure [40], it seemed possible that the inhibitory effects of C75 on feeding and energy expenditure are mediated by its suppressive action on ghrelin production. To test this hypothesis, we determined the effects of C75 on feeding, metabolism, sleep and motor activity in ghrelin receptor deficient mice.

## Methods

### a. Animals

Male, 5–6 months old ghrelin receptor KO (originally named as GHSR −/− mice, [41]) and wild-type (WT) mice were used in the experiments. Breeding pairs of ghrelin receptor KO and WT mice with a C57BL6J/129SvEv genetic background, backcrossed to C57BL6J for 10 generations, were generated and given as a generous gift by Drs. Roy G. Smith and Yuxiang Sun at Baylor College of Medicine (Houston, TX), and further bred at Washington State University. Each mouse used in the experiments was genotyped (Transnetyx, Cordova, TN). Procedures were carried out in accordance with the recommendations in the Guide for the Care and Use of Laboratory Animals of the National Institutes of Health. Protocol (ASAF # 3948) was approved by the Institutional Animal Care and Use Committee at the Washington State University.

### b. Experiment 1: The effects of C75 on sleep-wake activity, body temperature, home cage activity and food intake

#### Surgery

The body weight of the WT and KO mice at the time of surgery was 32.2±0.6 g and 32.8±0.7 g, respectively. During surgery, mice were anesthetized with intraperitoneal (ip) injection of ketamine-xylazine mixture (87 and 13 mg/kg, respectively), and all efforts were made to minimize suffering. The animals were implanted with cortical electroencephalographic (EEG) electrodes, placed over the frontal and parietal cortices, and electromyographic (EMG) electrodes in the dorsal neck muscles. The EEG and EMG electrodes were connected to a pedestal, which was fixed to the skull with dental cement. Temperature-sensitive transmitters were implanted in the abdominal cavity for telemetry recordings. Mice were allowed to recover from surgery for at least 2 weeks before baseline recordings started. During the recovery and experimental periods, all mice were housed in individual recording cages located in a sound-attenuated environmental chamber at a constant temperature (30±1°C) and controlled light–dark cycles (12–12 h, lights on: 4 am). Food and water were available *ad libitum* throughout the experiments. The animals were fed with regular lab chow (Harlan Teklad, Product No. 8460), in which fat, proteins, and carbohydrates provided 17%, 29%, and 54% of calories, respectively.

#### Sleep-wake recordings and analyses

Recording cables connected the animals to commutators, which were further routed to Grass Model15 Neurodata amplifier system (Grass Instrument Division of Astro-Med, Inc., West Warwick, RI). The high-pass and low-pass filters for EEG signals were 0.5 and 30.0 Hz, respectively. The EMG signals were filtered with low and high cut-off frequencies at 100 and 10,000 Hz, respectively. The outputs from the amplifiers were fed into an analog-digital converter (digitized at 256 Hz) and collected by computer (SleepWave software, Biosoft Studio, Hersey, PA). Sleep-wake states were scored visually off-line in 10-s segments according to the following criteria. Non-rapid-eye-movement sleep (NREMS): high-voltage EEG delta waves (0.5–4 Hz) and decreased muscle tone; rapid-eye-movement sleep (REMS): predominant EEG theta activity (6–8 Hz) and lack of muscle tone with occasional muscle twitches; wakefulness (W): low-voltage EEG activity, and varying levels of increased muscle activities. Time spent in W, NREMS and REMS was calculated in 2- and 12-h blocks. EEG power data from each artifact free 10-s segment were subjected to off-line spectral analysis by fast Fourier transformation. EEG power data in the range of 0.5 to 4.0 Hz during NREMS were used to compute EEG slow-wave activity (SWA). EEG SWA data were normalized for each animal by using the average EEG SWA across 24 h on the baseline day as 100. The results were averaged in 2-h bins.

#### Telemetry recordings

Core body temperature and locomotor activity were recorded by Mini Mitter telemetry system (Philips Respironics, Bend, OR). Temperature and activity values were collected every 1 and 10 min, respectively, throughout the experiment and were averaged into 2- and 12-h time blocks. Activity values were normalized for each animal by using the average activity across 24 h on the baseline day as 100.

#### Experimental procedures

After the recovery from surgeries, the mice were connected to recording cables and were given daily ip injections of saline for a 7-day habituation period. On the first experimental day (baseline day), the animals were injected with vehicle (ip, 1 ml/100 g body weight). On the following day, C75 (Sigma-Aldrich, St. Louis, MO; dissolved in RPMI 1640) was injected ip in the same volume. Three doses of C75 were tested on separate groups of WT and ghrelin receptor KO mice: 7.5 mg/kg (n = 8 for both WT and KO), 15 mg/kg (n = 10 for both WT and KO) and 30 mg/kg (n = 6 for WT and n = 8 for KO). The injections were performed during the last 15 min of the light phase. Recordings started from dark onset and continued for 24 h. Due to the malfunction of one transmitter, body temperature and activity data were collected only from 7 KO mice in the 7.5 mg/kg group. Due to the occurrence of EEG artifacts, one KO animal was excluded from the SWA analysis in the 30 mg/kg group. Each day, the animals received pre-weighed food pellets in their cage right after the injections. The leftovers were collected and measured 24 h later and daily food intake was calculated.

### c. Experiment 2: The effects of C75 on oxygen uptake, CO_2_ production, locomotor activity and food intake

In separate groups of mice (n = 5 for WT and n = 8 for ghrelin KO), oxygen consumption (VO_2_, ml/kg/h) and carbon dioxide production (VCO_2_, ml/kg/h) were measured with indirect calorimetry (Oxymax System, Columbus Instruments, Columbus, OH). Simultaneously, food intake (g/kg/h) and locomotor activity were determined. Locomotor activity was measured by infrared photocell sensors installed horizontally along the X- and Y-axes of the calorimetry cage. The sensors provided 1.27 cm beam spacing, 16 infrared beams intersecting the chamber in the X- and 8 beams in the Y-axis. The beam diameter was 0.32 cm. All four measurements were taken every 10 min and the data were collapsed into 1-h and 2-h bins. Respiratory exchange ratio (RER; VCO_2_/VO_2_) was calculated. The animals were habituated to the calorimetric cages for four days before the experiments. On the fifth day, mice were injected with vehicle (ip, 1 ml/100 g) and on the following day, they received 15 mg/kg C75, ip in the same volume. The injections were performed during the last 15 min of the light phase. Recordings started at light onset and continued for 24 h after each treatment. In 2 WT and 5 KO mice, food intake was measured for one additional day.

### d. Statistics

NREMS, REMS, W, SWA, average body temperature and motor activity in Experiment 1 and VO_2_, RER, food intake and activity in Experiment 2 were calculated in 2-h blocks. Three-way mixed ANOVA was performed separately for each dose of C75 across 24 h (independent measure: genotype, repeated measures: time and treatment). Daily food intake was analyzed by using two-way mixed ANOVA (independent measure: genotype, repeated measure: treatment) in Experiment 1. When ANOVA indicated significant effects, *post hoc* paired t-tests (for repeated measures) or Student's t-tests (for independent measures) were performed for all experiments. For Experiment 2, correlation between the effects of C75 on feeding and activity, on feeding and VO_2_ and on activity and VO_2_ was determined by using Pearson correlation test. For this, differences between treatment day and baseline were calculated in 1-h blocks and data were pooled from both genotypes.

## Results

### Experiment 1. The effects of C75 on the sleep-wake activity, body temperature, motor activity and food intake in ghrelin receptor KO and WT mice

C75 elicited characteristic behavioral responses in both genotypes. The animals became inactive after the injections and tended to lie quietly on the cage floor. These responses were particularly pronounced after the injection of the middle and the high doses of C75. At the end of the 24-h recording period we observed that the stool of C75-injected mice had been loose, often fluid-state, diarrhea-like. Although this phenomenon was not quantified, we did not notice any gross difference between the stool of WT and ghrelin receptor KO animals.

**Figure 1:**
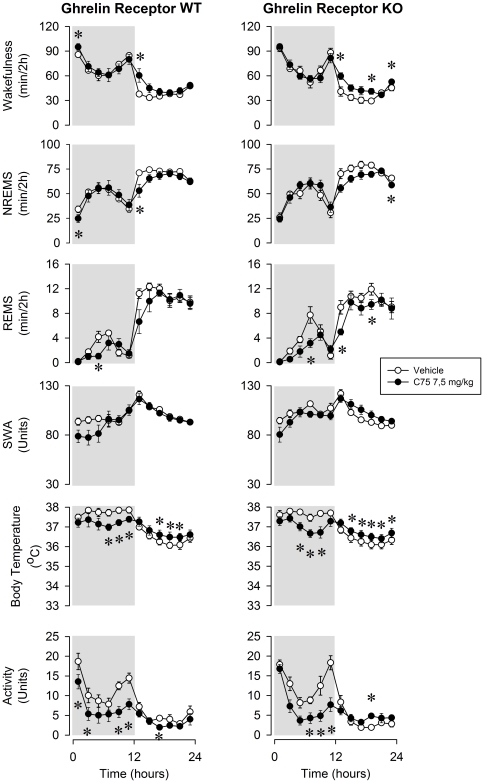
Wakefulness, non-rapid-eye-movement sleep (NREMS), rapid-eye-movement sleep (REMS), slow-wave activity of the electroencephalogram (SWA), body temperature and locomotor activity in ghrelin receptor wild-type (WT) and knockout mice (KO). Open symbols: vehicle injection, solid symbols: 7.5 mg/kg C75 intraperitoneally (ip). Data were calculated in 2-h time blocks. Time “0”: time of the injections. Activity is expressed as the percent of the total 24-h activity on the vehicle day. Dark shaded areas: dark period. Error bars: standard error. Asterisks denote significant differences between control and treatment days (*post hoc* paired t-test).

**Figure 2:**
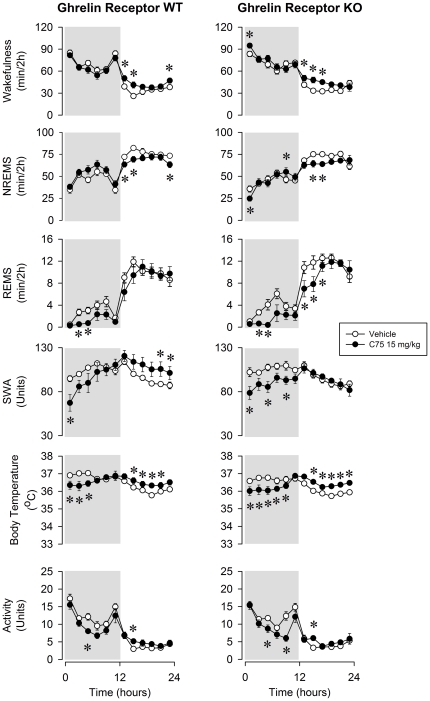
Wakefulness, NREMS, REMS, EEG SWA, body temperature and locomotor activity in ghrelin WT and KO mice after vehicle injection (open symbols) and 15 mg/kg C75 (solid symbols) administration. See legends to Figure 1 for details.

**Figure 3:**
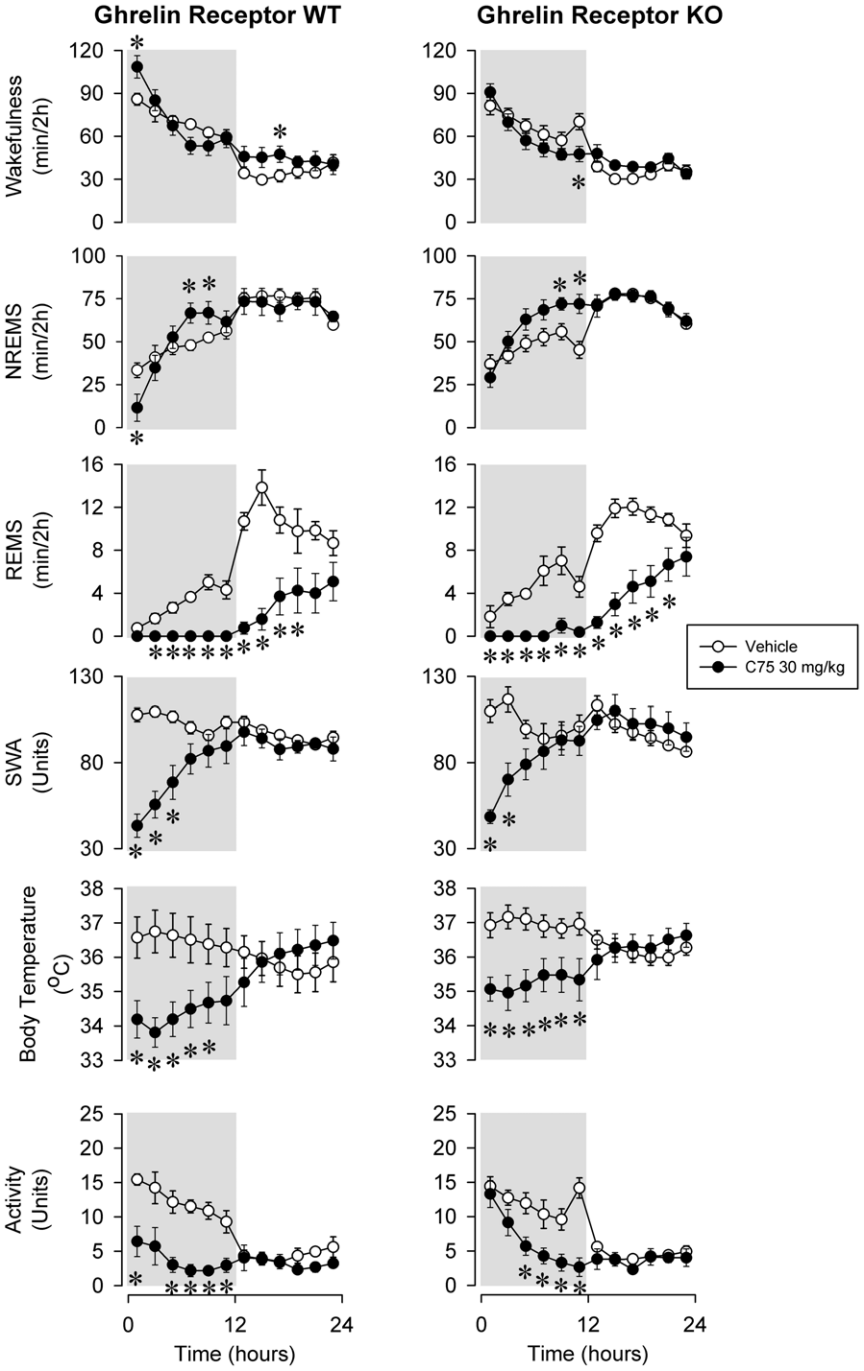
Wakefulness, NREMS, REMS, EEG SWA, body temperature and locomotor activity in ghrelin WT and KO mice after vehicle injection (open symbols) and 30 mg/kg C75 (solid symbols) administration. See legends to Figure 1 for details.

The most striking effect of C75 on sleep-wake activity was the dose-dependent, prolonged and robust suppression of REMS (Figures 1, 2 and 3). In WT mice, across the 24-h recording period, REMS was 9.3±3.0, 17.0±9.3% and 76.5±9.7% below baseline after the low, middle and high dose of C75, respectively. REMS was completely eliminated for 6 h after the middle and for 12 h after the highest dose of C75. Wakefulness was significantly elevated in the first 2-h time period after the treatment and subsequently, after a delay of ∼12 h during the light phase. Inverse changes in NREMS mirrored the wakefulness effects. Similar sleep changes were observed in ghrelin receptor KO animals. The daily amount of REMS was suppressed by 19.5±5.8, 24.7±6.1 and 66.8±9.6% after the injection of the three doses of C75. The lack of significant genotype effects or genotype×treatment interactions in ANOVA indicates that the sleep-wake responses of ghrelin receptor KO animals were not different from those of WTs (Table 1).

**Table 1:** The effects of C75 on sleep, wakefulness and slow-wave activity (SWA) of the electroencephalogram: statistical results.

	7.5 mg/kg			15 mg/kg			30 mg/kg		
	df	F	p	df	F	p	df	F	p
***NREMS***									
Treatm.	1,14	2.5	n.s.	1,18	1.5	n.s.	1,12	1.8	n.s.
Gen.	1,14	0.3	n.s.	1,18	2.1	n.s.	1,12	0.4	n.s.
Treatm.×Gen.	1,14	0.1	n.s.	1,18	0.7	n.s.	1,12	1.4	n.s.
Time×Gen.	11,154	0.8	n.s.	11,198	1.4	n.s.	11,132	1.4	n.s.
Treatm.×Time	11,154	3.2	P<0.05	11,198	4.3	P<0.05	11,132	7.6	P<0.05
***REMS***									
Treatm.	1,14	13.9	P<0.05	1,18	14.6	P<0.05	1,12	86.9	P<0.05
Gen.	1,14	0.1	n.s.	1,18	1.5	n.s.	1,12	2.3	n.s.
Treatm.×Gen.	1,14	0.5	n.s.	1,18	0.9	n.s.	1,12	0.0	n.s.
Time×Gen.	11,154	2.9	P<0.05	11,198	1.1	n.s.	11,132	0.6	n.s.
Treatm.×Time	11,154	3.0	P<0.05	11,198	3.5	P<0.05	11,132	9.3	P<0.05
***Wakefulness***									
Treatm.	1,14	4.2	n.s.	1,18	6.4	P<0.05	1,12	0.8	n.s.
Gen.	1,14	0.2	n.s.	1,18	1.9	n.s.	1,12	0.9	n.s.
Treatm.×Gen.	1,14	0.0	n.s.	1,18	1.6	n.s.	1,12	1.9	n.s.
Time×Gen.	11,154	1.0	n.s.	11,198	2.6	n.s.	11,132	1.2	n.s.
Treatm.×Time	11,154	3.2	P<0.05	11,198	3.4	P<0.05	11,132	6.6	P<0.05
***SWA***									
Treatm.	1,14	1.2	n.s.	1,18	0.8	n.s.	1,11	10.1	P<0.05
Gen.	1,14	1.4	n.s.	1,18	1.7	n.s.	1,11	1.0	n.s.
Treatm.×Gen.	1,14	1.4	n.s.	1,18	1.7	n.s.	1,11	1.0	n.s.
Time×Gen.	11,154	3.0	P<0.05	11,198	1.6	n.s.	11,121	0.6	n.s.
Treatm.×Time	11,154	2.9	P<0.05	11,198	8.9	P<0.05	11,121	19.5	P<0.05

Treatm.: Treatment; Gen.: Genotype; NREMS: non-rapid-eye-movement sleep; REMS: rapid-eye-movement sleep; n.s.: not significant, df: degrees of freedom.

In WT mice, C75 treatment elicited dose-dependent significant suppressions of the EEG SWA (Figures 1, 2 and 3). In response to 30 mg/kg C75, SWA remained 40–80% below baseline levels for a period of 6 h. FAS inhibitor treatment elicited a robust and long-lasting hypothermic response. In response to the highest dose of C75, the hypothermia lasted for 10–12 h with the peak response of a ∼2.5°C drop in temperature. Body temperatures then gradually returned to baseline by the beginning of the light period. The KO animals showed very similar SWA and temperature responses; the effects of C75 did not differ significantly in the two genotypes (Table 1).

**Figure 4:**
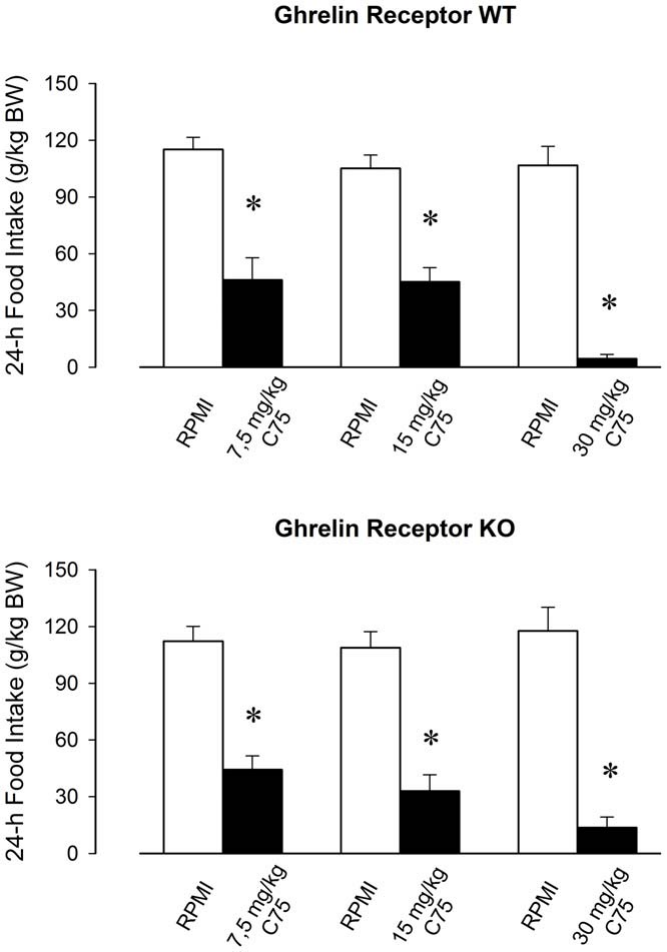
Daily food intake after vehicle (white bars) and 7.5, 15 and 30 mg/kg C75 (black bars) injections in ghrelin receptor WT and KO mice. Asterisks denote significant differences between control and treatment days (paired t-test). All doses of C75 significantly suppressed 24-h food intake. There was no significant difference in the effect of C75 between the two genotypes.

Activities of both WT and ghrelin receptor KO mice were significantly reduced by C75 (Figures 1,2 and 3). These effects were mainly confined to the first 12-h period when spontaneous activity is the highest. In response to 30 mg/kg C75, activity was suppressed by 69.3±8.6% in WT and 48.0±9.9% in KO animals in the dark phase. C75 dose-dependently inhibited 24-h food intake (Figure 4). WT mice ate 4.9±2.8% and KO mice 12.7±4.6% of the baseline food amounts following the highest C75 dose. There were no significant differences in the motor activity- or feeding-suppressing effects of C75 between the two genotypes (Table 2).

**Table 2:** The effects of C75 on body temperature, activity and feeding: statistical results.

	7.5 mg/kg			15 mg/kg			30 mg/kg		
	df	F	p	df	F	p	df	F	p
***Body Temperature***									
Treatm.	1,13	4.3	n.s.	1,18	0.3	n.s.	1,12	14.1	P<0.05
Gen.	1,13	0.1	n.s.	1,18	2.1	n.s.	1,12	0.8	n.s.
Treatm.×Gen.	1,13	0.0	n.s.	1,18	0.0	n.s.	1,12	0.1	n.s.
Time×Gen.	11,143	0.6	n.s.	11,198	0.9	n.s.	11,132	1.1	n.s.
Treatm.×Time	11,143	13.7	P<0.05	11,198	20.3	P<0.05	11,132	51.6	P<0.05
***Activity***									
Treatm.	1,13	34.9	P<0.05	1,18	9.4	P<0.05	1,12	37.7	P<0.05
Gen.	1,13	0.7	n.s.	1,18	0.0	n.s.	1,12	1.3	n.s.
Treatm.×Gen.	1,13	0.7	n.s.	1,18	0.0	n.s.	1,12	1.3	n.s.
Time×Gen.	11,143	0.7	n.s.	11,198	0.5	n.s.	11,132	0.7	n.s.
Treatm.×Time	11,143	6.8	P<0.05	11,198	5.9	P<0.05	11,132	8.5	P<0.05
***Feeding***									
Treatm.	1,14	84.9	P<0.05	1,18	81.2	P<0.05	1,12	120.8	P<0.05
Gen.	1,14	0.0	n.s.	1,18	0.3	n.s.	1,12	0.7	n.s.
Treatm.×Gen.	1,14	0.0	n.s.	1,18	0.7	n.s.	1,12	0.0	n.s.

### Experiment 2. The effects of C75 on VO_2_, RER, feeding and activity in ghrelin receptor KO and WT mice

**Figure 5:**
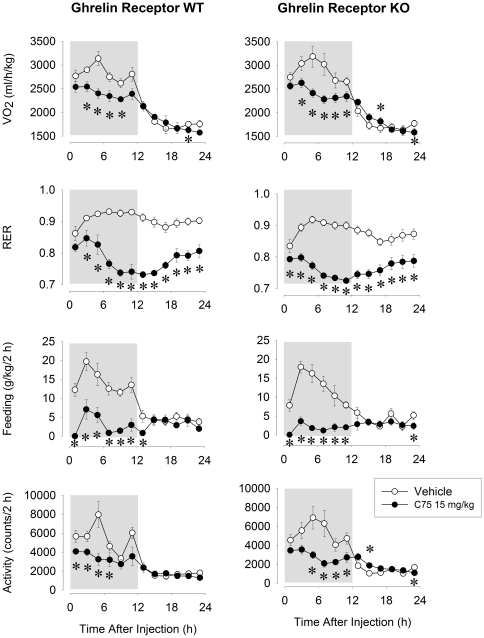
Oxygen consumption (VO_2_), respirator exchange ratio (RER), food intake and motor activity after the ip injection vehicle (open symbols) and 15 mg/kg C75 (solid symbols) in ghrelin receptor WT and KO mice. Data were calculated in 2-h time blocks. Activity is expressed as absolute values. Dark shaded areas: dark period. Error bars: standard error. Asterisks denote significant differences between control and treatment days (*post hoc* paired t-test).

In separate groups of KO and WT mice, we tested the effects of 15 mg/kg C75 on VO_2_, RER, feeding and activity. There were no significant differences in VO_2_, RER, feeding and activity between the two genotypes under normal, baseline conditions (Figure 5, Table 3). Spontaneous feeding and motor activities showed typical nocturnal pattern with 75–76% of feeding and 76–79% of total daily activity taking place during the dark phase. Similar nocturnal patterns were observed in VO_2_, with 61–62% of the daily oxygen consumption occurring during the dark period. RER slightly increased during the dark followed by a decline in the light period.

**Table 3:** The effects of C75 on oxygen uptake (VO_2_), respiratory exchange ratio (RER), feeding and activity: statistical results.

	df	F	p
***VO_2_***			
Treatm.	1,11	26.7	P<0.05
Gen.	1,11	0.0	n.s.
Treatm.×Gen.	1,11	0.0	n.s.
Time×Gen.	11,121	0.4	n.s.
Treatm.×Time	11,121	12.4	P<0.05
***RER***			
Treatm.	1,11	136.3	P<0.05
Gen.	1,11	2.8	n.s.
Treatm.×Gen.	1,11	0.2	n.s.
Time×Gen.	11,121	0.5	n.s.
Treatm.×Time	11,121	17.3	P<0.05
***Feeding***			
Treatm.	1,11	114.3	P<0.05
Gen.	1,11	1.3	n.s.
Treatm.×Gen.	1,11	0.2	n.s.
Time×Gen.	11,121	1.3	n.s.
Treatm.×Time	11,121	18.0	P<0.05
***Activity***			
Treatm.	1,11	24.4	P<0.05
Gen.	1,11	1.2	n.s.
Treatm.×Gen.	1,11	0.0	n.s.
Time×Gen.	11,121	0.5	n.s.
Treatm.×Time	11,121	9.5	P<0.05

Consistent with our findings in Experiment 1, 15 mg/kg C75 significantly reduced motor activity and feeding during the dark period in both WT and KO animals; there was no difference in these effects between the two genotypes (Figure 5, Table 3). During the light, *i.e.*, 13–24 h after C75 treatment, feeding did not differ significantly from baseline. In 2 WT and 5 KO mice, we recorded feeding on the subsequent recovery day. Pooled data from the 7 animals showed that feeding was still suppressed by ∼30% below baseline during the dark phase of the recovery day, i.e., 24–36 h after C75 injection (data not shown).

**Figure 6:**
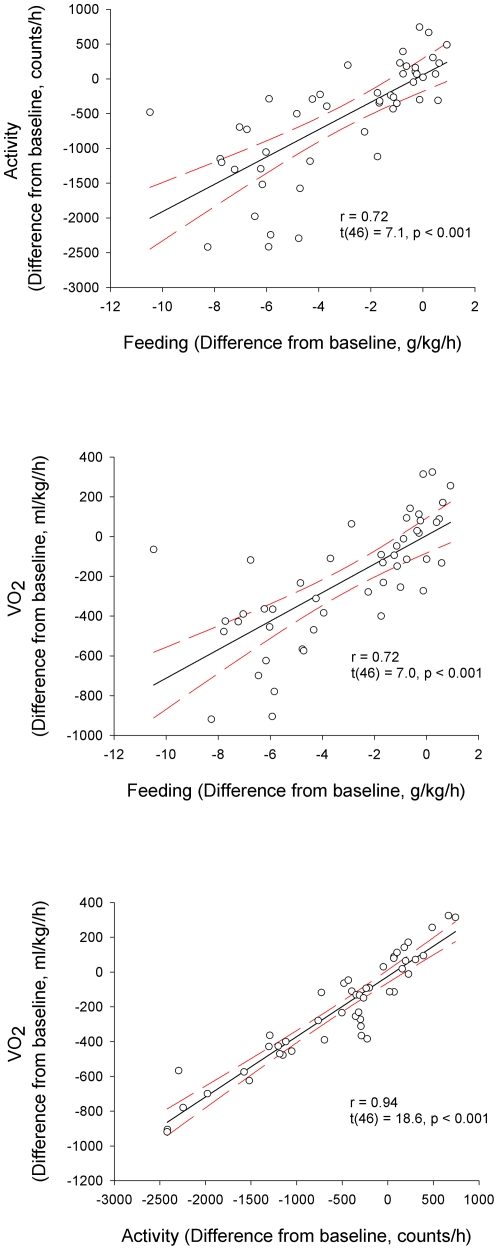
Correlation between the effects of 15 mg/kg C75 on feeding and activity (upper panel), on feeding and VO_2_ (middle panel) and on activity and VO_2_ (bottom panel). Pearson correlation was calculated from the 1-h differences between C75 and vehicle day. Data from both genotypes are pooled. Dashed lines: 95% confidence intervals.

After C75 injection, VO_2_ was suppressed by 14.5±2.3% in WTs and by 15.5±2.6% in KO mice during the night. The values returned to baseline by the light period. RER was decreased throughout the entire 24-h postinjection period. On the baseline day, average 24-h RER values were 0.91±0.01 and 0.88±0.01 in WT and KO mice, respectively, while after C75 treatment, these values were suppressed to 0.78±0.01 and 0.76±0.01. The lack of significant genotype effect or genotype×treatment interaction indicates that the feeding, activity, VO_2_ and RER responses to C75 were the same in both WT and KO mice (Table 3). Since there was no significant difference in the effects of C75 between the two genotypes, we pooled data from the two groups for correlation analysis. We found significant correlation between the effects of C75 on feeding and activity, the effects on feeding and VO_2_ and the effects on activity and VO_2_ (Figure 6, Pearson correlation test, p<0.001 for each).

## Discussion

Our major finding is that systemic injection of C75 suppresses motor activity, REMS, and SWA of the EEG in both normal and ghrelin receptor KO mice. These behavioral and sleep effects are accompanied by decreases in VO_2_, body temperature and RER. We confirm our and others' previous findings that spontaneous sleep-wake activity [22], motor activity [22], [42], [43] and food intake on standard laboratory diet [41], [44] are not affected in ghrelin receptor KO mice. Our results also confirm that C75 elicits robust dose-dependent inhibition of 24-h food intake [37], [39]. The effects of C75 on the daily rhythm of feeding have not been reported before. We show that C75 abolished the diurnal rhythm of feeding. Night-time food intake was decreased to the level normally seen during the day, the rest period in mice. The effects of C75 on motor activity have not been quantified previously. In two studies, no “gross changes” in the activity of mice [37] or no “obvious motoric effects” in rats [39] were observed after systemic injection of C75. In the present study, we quantified the effects of C75 on spontaneous motor activity in two separate sets of experiments, by using two different methods. We found that spontaneous motor activity was decreased after ip injection of C75 as measured both by the movement of implanted transponders over a horizontal receiver in Experiment 1 and by the interruption of infrared beams in Experiment 2.

The mechanism of the anorectic effects of C75 is not well understood. It is possible that C75 stimulates satiety circuits and/or suppresses orexigenic mechanisms or inhibits feeding by eliciting visceral illness. In the present study, we tested the role of orexinergic mechanisms in the effects of C75 by using ghrelin receptor KO mice. Ghrelin plays a central role in physiologic orexigenic mechanisms (reviewed in [23]) and stimulates motor activity [31], [45]. It has been postulated that increased behavioral activity and feeding in the beginning of the dark period are due to the activation of ghrelin circuits in the brain of nocturnal rodents [31]. C75 is known to inhibit ghrelin secretion in mice [36] thus it seemed possible that suppressed ghrelin secretion may account for both the anorectic and motor activity-suppressing effects of C75. In this case, intact ghrelin receptor-mediated signaling would be required for the manifestations of these actions, *i.e.*, C75 would not affect eating and activity in animals that lack ghrelin receptors. The finding that C75-induced anorexia and motoric inhibition are not attenuated in ghrelin receptor KO mice indicates that these effects of C75 are unrelated to the function of ghrelin receptors.

We found significant correlation between the anorectic and motor activity effects of C75. An overall decrease in motor activity may, in theory, lead to decreased feeding. Although causality cannot be determined with certainty in the present study, it seems unlikely that the anorectic effects are exclusively due to the general suppression of motor activity. First, while the overall time-courses correlate significantly, there is a meaningful dissociation between the motor activity- and feeding-suppressive effects of C75. For example, in the ghrelin receptor KO mice, motor activity was unchanged in the first 4 h after C75 treatment but feeding was already suppressed to ∼20% of the baseline. Second, if it is the decreased motor activity that interferes with normal feeding behavior after C75 injection then one would expect that the drive for food, *i.e.*, hunger, progressively increases during the dark. Hunger induced by food restriction is accompanied by characteristic changes in sleep-wake activity and c-Fos activation pattern in the brain. Overnight food deprivation results in a ∼24% increase in waking time during the dark in mice [22]. This arousal response was not observed after C75 injection supporting the notion that the animals were not hungry, in spite of the fact that they ate less than 18% of their normal food amount during the dark period. Also, food deprivation stimulates c-Fos expression in orexigenic brain structures such as the paraventricular nucleus (PVN), ARC and LH, but systemic C75 treatment fails to elicit similar activation pattern [46].

A possible explanation for the decreased feeding after C75 injection is that C75 elicits a satiety-like state. The sleep findings, however, do not support this notion. Both naturally occurring satiety that follows feeding [1]–[4] as well as injection of satiety-inducing hormones such as cholecystokinin [11], [12] lead to increases in sleep. In our study, however, C75 induced dose-dependent and long-lasting suppression of REMS. Thus the sleep phenotype after C75 treatment does not match fasting or satiated conditions but shows close similarity to the sleep pattern described in visceral pain models. Visceral illness elicited by LiCl injections is accompanied by transient increase in wakefulness followed by long-lasting suppression of REMS [47], [48]. An ip bolus injection of LiCl causes significant increase in the latency and a significant reduction in the occurrence of REM sleep in the immediate hours following the injection. In contrast, NREM sleep occurrence is only slightly affected by lithium administration. LiCl treatment significantly reduces the relative delta power of the EEG after LiCl treatment [49]. We also observed the suppression of EEG SWA, *i.e.* delta waves, after C75 administration. Furthermore, LiCl treatment leads to behavioral inactivity and causes rats to lie quietly on the floor of the cage and elicits diarrhea [50]. These sleep and behavioral effects are strikingly similar to those we found in response to C75 treatment. We and others [51] also observed soft, diarrhea-like stool of the animals after systemic injection of C75. The pattern of brain c-Fos induction after C75 treatment is also consistent with visceral illness. Systemic injection of 10 or 30 mg/kg C75 induces intensive c-Fos activation in the PVN and the nucleus tractus solitarius/area postrema (NTS/AP) 1–2 h after the injection [20], [46]. Similarly, ip injection of malaise-inducing doses of LiCl causes c-fos activation in the hypothalamic PVN and in the brainstem NTS [52]–[54]. Systemic injection of C75 produces conditioned taste aversion further supporting the notion of visceral illness [39], [55].

In agreement with previous reports [44], [56], [57], there was no difference in the baseline energy expenditure or RER between ghrelin receptor KO and WT mice. Systemic bolus injection of C75 suppressed energy expenditure as reported earlier [38], [39] and also decreased RER. There was no difference in these responses between the two genotypes indicating that ghrelin signaling is not required for the metabolic actions of C75. Suppressed energy expenditure and RER are consistent with the state of energy conservation and a shift to lipid catabolism, typical metabolic responses to fasting. It is likely that these responses to C75 are also secondary to suppressed feeding. The strong correlation between the time course of the anorectic effect and the suppression of energy expenditure further support this notion. In one study [37], but not in others [46], [58], C75 treated animals lost more weight than the pair-fed controls leading to the speculation that C75 has an additional, direct energy expenditure-stimulating effect [38]. The observation, however, that the treatment causes diarrhea leads to an alternative explanation for the increased weight loss after C75 administration.

C75 is a potent inhibitor of FAS *in vitro*. It has been proposed that weight loss-inducing and food intake-suppressive effects of C75 are related to the suppression of FAS activity in the hypothalamus [37]. According to this hypothesis, FAS inhibition-induced increases in malonyl-CoA in hypothalamic neurons provide a signal that leads to the activation of feeding-suppressive mechanisms. The efficiency of systemic C75 treatment to suppress brain FAS activity is, however, questionable. Systemic injection of 30 mg/kg C75 does not affect hypothalamic FAS enzyme activity [20], [59], therefore it can be ruled out that the observed sleep, activity, body temperature and metabolic effects observed in our experiments are related to the inhibition of FAS activity in the brain. Furthermore, the plasma levels of C75 after administering 10 or 30 mg/kg ip reach less than 1% of the concentration needed for FAS inhibition *in vitro* [20]. C75, however, has significant anorectic, motor activity-inhibiting and metabolic effect in this dose range suggesting that these actions are unrelated to the inhibition of brain FAS. The most parsimonious explanation for the observed actions is that they are due to the aversive actions of C75. As a result of visceral illness, feeding and motor activity are suppressed. Decreased eating and activity lead to suppressed energy expenditure with the concomitant decrease in body temperature and to the shift from carbohydrate to lipid utilization manifested as decreased RER. The disrupted sleep pattern likely reflects the aversive effects. Since C75 has sparked interest for its potential use in body weight reduction and cancer therapy, its effects on sleep, activity and metabolism need to be considered also in this context. In conclusion, we demonstrated that systemic injection of C75 induces long-lasting decreases in sleep, motor activity, feeding, VO_2_ and RER. It is unlikely that these actions are due to the effects of C75 on brain FAS or the ghrelin system.
